# Innovative Application of SERS in Food Quality and Safety: A Brief Review of Recent Trends

**DOI:** 10.3390/foods11142097

**Published:** 2022-07-14

**Authors:** Meng-Lei Xu, Yu Gao, Xiao-Xia Han, Bing Zhao

**Affiliations:** 1 State Key Laboratory of Supramolecular Structure and Materials, College of Chemistry, Jilin University, Changchun 130012, China; xumenglei@jlu.edu.cn (M.-L.X.); hanxiaoxia@jlu.edu.cn (X.-X.H.); 2 College of Food Science and Engineering, Jilin University, Changchun 130062, China; 3 College of Plant Protection, Jilin Agricultural University, Changchun 130118, China; gaothrips@jlau.edu.cn

**Keywords:** surface-enhanced Raman scattering (SERS), food quality, safety, authenticity, poisoning, contaminant, genetically modified food (GMF), insects food, semiconductor, coronavirus disease 2019 (COVID-19)

## Abstract

Innovative application of surface-enhanced Raman scattering (SERS) for rapid and nondestructive analyses has been gaining increasing attention for food safety and quality. SERS is based on inelastic scattering enhancement from molecules located near nanostructured metallic surfaces and has many advantages, including ultrasensitive detection and simple protocols. Current SERS-based quality analysis contains composition and structural information that can be used to establish an electronic file of the food samples for subsequent reference and traceability. SERS is a promising technique for the detection of chemical, biological, and harmful metal contaminants, as well as for food poisoning, and allergen identification using label-free or label-based methods, based on metals and semiconductors as substrates. Recognition elements, including immunosensors, aptasensors, or molecularly imprinted polymers, can be linked to SERS tags to specifically identify targeted contaminants and perform authenticity analysis. Herein, we highlight recent studies on SERS-based quality and safety analysis for different foods categories spanning the whole food chain, ‘from farm to table’ and processing, genetically modified food, and novel foods. Moreover, SERS detection is a potential tool that ensures food safety in an easy, rapid, reliable, and nondestructive manner during the COVID-19 pandemic.

## 1. Introduction

Food quality and safety are prominent components of food science research [1]. In this context, it is essential that reliable systems are developed to detect, eliminate, and control the risks posed by hazardous substances [2]. Surface-enhanced Raman scattering (SERS) is a powerful molecular spectroscopy technique, based on inelastic scattering enhancement from molecules located near the nanostructured metallic surfaces, significantly differing from the gold standard methods, including chromatography and mass spectroscopy [3]. Over the past four decades, the great potential of SERS for the rapid detection of trace chemicals has been demonstrated. Excellent review papers have been published that focus on different perspectives of SERS technology, including its mechanisms, semiconductor SERS substrates, and the applications in food safety and quality, which may be of interest to different readers [4]. Semiconductor materials have more controllable properties than metals, including their band gap, photoluminescence, stability, and degradation resistance [5]. Our research group has also published related reviews and articles that provide details regarding SERS for food safety applications [6].

**Figure 1:**
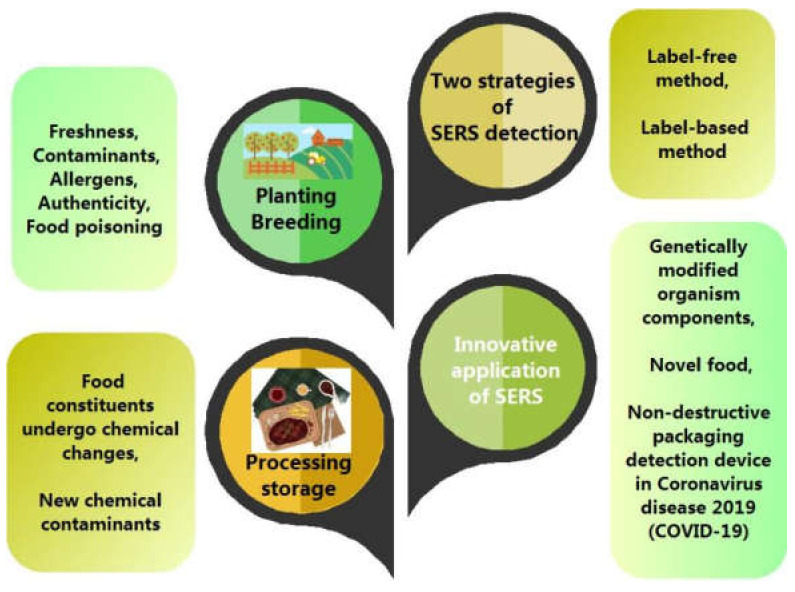
SERS applications in food quality and safety.

SERS technology is still not completely ready for use as a routine analytical tool to solve real-world food analysis problems, but it is beginning to be applied, especially for conducting ‘Recognition’ to ‘Behavior analysis’ of both food ingredients and pollutants. This can cover the whole food chain, including food planting, breeding, packing, storage, transportation, and processing [7]. Foods can be classified into the following groups: grains; oil-producing plants; vegetables; fruits; nuts; sugars; beverages; edible fungi; flavorings; medicinal plants; and foods of animal origin. The food matrix complexity and different processing technologies produce different concerns regarding the quality and safety of each food type. Herein, we summarize the innovative application of SERS in food quality and safety, based on food classification (Figure 1). 

## 2. SERS Applications in Food Basal Components and Freshness

Food is composed of complex materials and the detection of specific ingredients is an important step. Basal food components include moisture, protein, oil, ash, reducing sugars, and other ingredients. However, the SERS methods are not yet widely available as a tool in food analytical laboratories, especially for the basal components. The analyte response can be increased to achieve an effective SERS signal [8] and the SERS data can be arranged in vector form, belonging to the first-order category. Particularly successful first-order calibrations include partial least-squares (PLS) and PLS regression (PLSR), that permit the quantitation of the selected analytes without knowing the chemical identity of the interfering species [9].

Two strategies to judge freshness include: (1) using the relationship between normally bright matter and Raman signals to judge freshness. In particular, for fruits and vegetables, their respective Raman assignments arise from glucose, carotene, and lycopene and can reflect freshness [10]; (2) monitoring specific marker molecules of corruption and deterioration. For example, ammonia and formaldehyde are spoilage indicators of fish that can be monitored [11]. The SERS methods can detect fructose and pectin in commercial apple juice and pear/apple pulp when combined with the wavelet denoising algorithm and the background subtraction method [12]. Thus, the Raman/SERS spectra may not directly determine the components that influence freshness, but can indirectly monitor the changes in basal components through spectral changes and detecting specific molecules (Table 1) [13]. The SERS spectra also showed that discrimination between products and manufacturers is possible through the fingerprint analysis [14]. For example, when coupled with PCA, the SERS method can be applied for white wine characterization, wherein the main spectral differences arise from adenine, carboxylic acids, and glutathione, with their ratios changing between wine types and producers [15].

Foods of an animal origin are often more highly processed than fruits and vegetables, necessitating on-site quality assessments because of their vulnerability to contamination and deterioration. For a qualitative analysis of foods of animal origin, Raman sensors can provide depth-profile information with regards to the purines, proteins, and lipids with repetitive regeneration. Meanwhile, spectral analysis algorithms can simplify the analytical data processing and improve the accuracy and robustness of spectral multivariate analysis [16]. The online monitoring of food freshness and internal ingredients is important, with Raman detection systems well-suited for this application. In addition, the structural information contained in the Raman spectra can be archived into electronic files corresponding to the specific food samples for subsequent reference.

## 3. SERS Application in “From Farm to Table” Foods

### 3.1. Chemical Contaminants and Toxin Detection by SERS

Chemical contaminants (e.g., chemical fertilizers, pesticides, veterinary drugs, and hormones) are major food safety issues [17], and many studies have reported comprehensive strategies for chemical contaminant detection [18,19]. Generally, foods can be categorized as liquid, solid, or solid and liquid mixtures, contaminants, or poisons distributed on the surface of these food types and can be detected by SERS [20]. This is usually accomplished using either label-free or label-based methods. The recognition elements of chemical contaminants and poison include probe molecules, modified substrates, and aptamers. Increasing efforts are expected to focus on developing novel SERS substrates and highly sensitive Raman reporters, including semiconductor nanomaterials and related composites (Table 2) [21,22,23,24].

The chemical contamination residues in different food categories detected by SERS methods can vary. For the pesticide residues as an example, more reports on beverages, fruits, and vegetables have been published than for other foods [25]. For foods of animal origin, chemical contaminants in the feed and veterinary drug residues and their metabolites may enter the food chain during breeding and raising. In addition to the base drugs, their matrix, transformation products, reaction products, and impurities of toxicological significance can significantly affect food safety [26]. The detection methods for chemical pollutants in foods of animal origin are similar to those used for vegetables and fruits, with the exception of the requirement for efficient lipid separation. Although most foods are solids or solid–liquid mixtures, few studies regarding the detection of contaminations inside solid foods have been reported. This is because an extraction process is necessary prior to analysis to increase the analyte concentration and improve the SERS response.

Beverages and wine are liquids that are very suitable for SERS detection without pretreatment to test food quality or safety. For food quality testing, SERS substances, such as Ag NPs, can react with the components in red or white wine, trapping the color and flavor components and yielding a specific SERS signal for the sensitive detection of certain components [27]. The chemical or biological contaminants in beverages and wine mainly include ethyl carbamate, fusel oil, aldehyde, manganese, aflatoxin, patulin toxin, and N-nitrosodimethylamine, which can be detected using the label-free or label-based SERS methods. In addition to the conventional chemical or biological contaminants, harmful gas residues can be produced during fermentation and beverage production, for example, sulfite residues/SO_2_ [28]. The SO_2_ binds to the tertiary amine group on the Ag nanofilm substrate to realize detection by spectral changes, instead of sensing in the solution phase [29]. A gas-diffusion microfluidic paper-based analytical device combined with SERS was also applied for sulfite determination in wine [30], allowing the target analyte to be detected in the gas phase.

### 3.2. Biological Contaminant Detection by SERS

#### 3.2.1. Mycotoxins Contaminants

In addition to chemical pollution, biological contaminants, including microorganisms and their toxic metabolites, viruses, parasites and their eggs, and vector insects, pose food safety risks. Among these, mold and mycotoxin contamination are the most common [31,32]. Unlike vegetables and fruits, grains and nuts require long-term storage facilities, and are more suitable for storage because of their lower water content. Current SERS detections are usually accomplished by label-free (direct) or label-based (indirect) methods. Although the mycotoxins are usually distributed on the surface of the solid or liquid food, extraction processes are necessary to yield accurate SERS results [33,34]. For label-based mycotoxin detection, the use of recognition elements for biological contaminants, such as antibody/immunoassays, aptamers, molecularly imprinted polymers (MIPs), linear polymer affinity agents (LPAA), and/or specific surface-modified nanomaterials, have been applied (Table 3) [35,36].

#### 3.2.2. Bacterial Contamination Detection by SERS

Biological contaminants are common in foods of animal origin. Label-free and label-based SERS methods can be applied equally here, but with slight differences [37]. *Bacillus*, a spore-forming bacterium, is commonly detected using a biomarker, such as calcium dipicolinate (CaDPA) or dipicolinic acid (DPA), associated with bacterial spores and can be detected by SERS [38]. The label-free SERS profiles of bacteria offer signatures of molecular structures, cellular compositions, and physiological states, and can be applied to discriminate between bacterial species or to distinguish between live and dead bacteria [39,40]. For the label-based SERS methods to detect bacterial contamination, recognition elements, such as antibodies, aptamers, or small-molecule ligands, must be cross-linked to the SERS tags to improve the specificity for the targeted bacterial pathogen (Table 4) [41,42].

### 3.3. Harmful Metal Contaminant Detection by SERS

Hg, Pb, Cd, As, Cr, Al, and F are commonly detected harmful elements in food. Their overall quantities and speciation are related to their accumulation capacity and biological toxicity. For SERS detection, these heavy metals are different from other chemical contaminants because they generally do not have chemical bonds that generate Raman scattering. Thus, only inorganic oxyanions and a few oxycations can be directly detected from their characteristic Raman bands. Label-free and label-based methods are suitable for detection, similar to other general chemical contaminants. For the label-free method, semiconductor substrates are prepared using metal oxides, which absorb metal cations [43,44]. For label-based methods, the formation of specific chemical bonds with heavy metal ions or extrinsic SERS probes are required for SERS detection. Generally, label-based methods for metal detection by SERS involves three types of recognition elements: Raman activity dye signal turn-on by the metal; Raman activity dye signal turn-off by the metal; or combination with other analytical techniques (Table 5) [45,46,47]. Therefore, novel SERS substrates should be developed to achieve a better selectivity and replace the requirement for separation.

### 3.4. Food Allergen Detection by SERS

Food allergies that are triggered by the ingestion of food protein antigens represent an important food safety issue. These reactions are mediated by an immunological mechanism involving specific IgE or cell-mediated mechanisms, with 90% of allergic reactions originating from milk and dairy products, eggs and egg products, fish and shellfish, peanuts, beans and bean products, nuts, and wheat [48,49]. Therefore, it is particularly important to develop sensitive and effective detection methods for these food allergens. This is similar to the biological pollutant detection method described above, and the label-based method (indirect) is an ideal detection strategy. An aptamer-based SERS assay for β-lactoglobulin in milk samples was developed, with a detection limit of 0.07 ng/mL [50]. Peroxidase-mimicking nanozyme-catalyzing signal amplification as part of a portable SERS immunoassay for the food allergy protein α-lactalbumin was developed, with a detection limit of 0.01 ng/mL [51]. The developed label-based methods, such as aptamer-based SERS assays and nanozyme-enhanced SERS immunoassays, demonstrate a broad potential for food allergen supervision (Table 6).

### 3.5. Food Poisoning Detection by SERS

Food poisoning is an important food safety problem and encompasses non-infectious polar and subacute diseases that occur after ingestion of food containing biological and chemical toxins. The types of poisoning can be divided into four categories: bacterial, fungal, or chemical food poisoning, toxic animals, and plant-based food poisoning. The first three types are caused by different chemical or biological contaminants and were discussed in previous chapters. Herein, we focus on the phytotoxins and toxic animal-related food poisoning.

The phytotoxins are a class of natural organic compounds with high biological activities and toxicities. Poisonous plants, such as toadstools, cassava, green beans, sprouted potatoes, and fresh day lily, can cause poisoning due to improper cooking. Legumes are the main source of toxin poisoning, with major toxins including plant erythrocyte lectin, trypsin inhibitor, saponin glycoside, and phytic acid. Grains, such as the Solanaceae and Liliaceae plants, can cause poisoning through their secondary metabolites. Toxic animal-based food poisoning is often caused by specific species or tissues, with common examples including the puffer fish, shellfish, animal thyroid, liver, tetrodotoxin, shellfish toxin, and ichthyocholao toxin [52]. Among these chemicals, phytic acid, tetrodotoxin, and saxitoxin have been detected using SERS methods [53,54,55]. These SERS toxin detection methods include both label-free and label-based strategies, similar to chemical contamination detection. SERS has great potential for the identification of trace food poisoning during or after food processing, because of the low toxic analyte concentration and complex food matrix structure. 

### 3.6. Food Authenticity Detection by SERS

The authenticity of food is another important research subject [56]. The adulteration of food products with cheaper materials for economic gain can pose serious health threats to consumers [57]. As vibrational spectroscopic techniques, Raman/SERS have the potential to fulfill the industrial need for food quality and authenticity analyses [56]. Two strategies to judge authenticity include: (1) combination with chemometrics to achieve the identification and quantification of food samples; (2) combination with specific target DNA-modified SERS substrates with a target-responsive Raman dye that can recognize the target (Table 7) [58,59]. The SERS biosensors are regarded as a universal platform for the on-site determination of food quality, authenticity, and safety [60].

## 4. SERS Applications in Processed Foods

### 4.1. Contaminants during Processing in Foods of Animal Origin

Notably, contaminants during the processing of foods of animal origin are chemicals generated when food constituents undergo chemical changes, including N-nitroso compounds, polycyclic aromatic hydrocarbons, acrylamide, and heterocyclic amines. There is a certain understanding of the formation mechanism of these compounds, but relevant reports remain sparse [61]. Many outstanding studies have developed SERS detection methods [62], but ultrasensitive, reliable, and facile detection technologies for processing contaminants and trace toxins in foods remain challenging to develop.

### 4.2. Oil Plants, Fats, and Fried Food

Edible oils can be classified based on their source as vegetable or animal oils, and their improper consumption and storage can endanger human health. Common oil quality and safety problems include rancidity, trans fatty acids produced during heating, and the presence of glucosinolate, erucic acid, and gossypol residues. These can be detected by confirming associated chemical substances. For example, malondialdehyde is a biomarker of lipid peroxidation that is traditionally associated with food rancidity [63]. However, it is worth noting that waste cooking oil from restaurants and street vendors, known as "recycled cooking oil”, contains significant amounts of endogenous pollutants. To this end, capsaicin was developed as a marker as part of a SERS methodology [64]. Moreover, during the processing of starch-rich grains, acrylamide is a major harmful chemical produced by frying and high-temperature baking [65]. Some of these chemical pollutants can be detected by SERS methods, but significant progress needs to be made to achieve widespread applicability.

### 4.3. Condiments

Soy sauce, vinegar, monosodium glutamate, sauces, and sugar are traditional condiments that can generate harmful substances during their production and fermentation. Chloropropanol is produced by hydrolyzing vegetable protein with hydrochloric acid and can be found in raw soy sauce, old soy sauce, and oyster sauce. However, a SERS detection method for chloropropanol has yet to be reported. Thus, more research is needed to rapidly detect these chemical substances in the condiment supply.

## 5. Genetically Modified Foods (GMFs) and Novel Foods

### 5.1. Genetically Modified Foods (GMFs)

The foods derived from genetically modified (GM) organisms are often referred to as GM foods (GMFs). The realization of GM technology in the agricultural system requires due diligence and in-depth analysis of the associated risks and/or benefits to multiple stakeholders on a case-to-case basis before commercialization [66]. For SERS detection, the rapid and simultaneous screening of multiple GM organism components (genes, promoters, codons, and terminators) in rice and soybean has been reported [67,68]. This DNA-conjugated SERS nano-tag detection strategy is similar to the authentication of foods of animal origin. This methodology can be applied in different detection scenarios, using the principle of similarity. Moreover, Raman is capable of extracting sample fingerprints and, in combination with chemometric tools, can discriminate transgenic corn with a predictive accuracy of 87.5% [69]. This also demonstrates the wide applicability of the SERS methodology (Table 8).

### 5.2. Novel Foods

‘Novel foods’ are newly developed, innovative food products produced using new technologies and production processes [70]. This includes meat alternatives and replacements produced from plant-based alternatives, as well as insects and fungi [71,72]. Current novel food regulations have adapted quickly to recognize the new food categories and add some to the existing food categories [73]. The qualitative and quantitative analytical data on substances hazardous to human health should also be provided [74]. SERS can provide a useful tool for recording full spectrum information, which is important for distinguishing the contaminants of unknown biological activity, including potential allergens. 

## 6. Application and Major Challenges of SERS Application in Coronavirus Disease 2019

Viruses are common biological contaminants in food, and foodborne viruses use food as carriers to cause human diseases, including those transmitted by the fecal–oral route (such as polio-, rota-, and coronaviruses) and livestock products (e.g., such as avian influenza, prion, foot-and-mouth disease). The proliferation and transmission of viruses have become a threat to biosecurity worldwide, as exemplified by the coronavirus disease 2019 (COVID-19) pandemic. The available evidence shows that the COVID-19 virus does not spread through food, but it can contaminate food. Thus, nondestructive and rapid detection in food packaging, matrix, and processing are particularly important [75]. The SERS materials modified with short-structured oligonucleotides (DNA aptamers) provide excellent specificity for SERS biosensors [76]. If a nondestructive packaging detection device is successfully developed and proven to detect target viruses, it could enable rapid and mass screening of COVID-19 in food samples and be used in future pandemic screening.

## 7. Conclusions

Emerging Raman spectroscopic techniques, including Raman spectroscopy, Raman mapping, and SERS for rapid and nondestructive analysis, have been developed as effective tools for both qualitative and quantitative analyses in most of the food categories. SERS is based on inelastic scattering enhancement from molecules located near nanostructured metallic surfaces. This mechanism differs from the gold standard methods, chromatography, mass spectroscopy, and other spectroscopy methods, featuring advantages such as ultrasensitive detection, simple protocols, lack of pretreatment, and reduced costs. Semiconductor-metal hybrid substrates have great potential for the highly robust SERS sensing of pesticides with high enhancement ability, good reproducibility, acceptable stability, and reusability. The component and structural information contained in the SERS/Raman spectra can generate an electronic file of the food samples for subsequent reference and food production traceability. Label-free and label-based methods can be used for chemical and biological contaminant detection. The recognition elements including immunosensors, aptasensors, and MIPs can be linked to SERS tags to improve the specificity for target contaminants and allergens in GMF or novel foods. Importantly, the contaminant detection and quality analysis in actual food samples can often differ from the detection in standard solutions, owing to the complexity of the food matrices. Most foods are solid or solid–liquid mixtures, with only a few studies on contamination inside solid foods. Thus, extraction processes are required for the effective detection in both quality and safety analyses. The SERS detection method is a promising tool that can ensure food safety in an easy, rapid, reliable, and nondestructive manner. Thus, despite the persisting limitations for ingredient and freshness determination resulting from technical difficulties and the complexity of the food sample matrix, the continuous development of Raman systems, nanomaterials, and spectral analysis algorithms will facilitate the transfer from laboratory to industry. Industrial application will span the entire food production process and benefit from the on-line application of this technology.

## Figures and Tables

**Table 1:** Strategies of food freshness and characterization judgment by SERS methods.

Strategies	Analysis	Algorithms	Ref.
Relationship between normally bright matter and Raman signal	Egg shell	Partial least-squares regression (PLSR)	[8]
	Oxidation process of nut oils	PLSR and Forest random PLSR (RF-PLSR)	[9]
	Citrus fruits	Raman coefficient of freshness (C_Fresh_)	[10]
	Red wine	PCA	[14]
	White wine	PCA	[15]
Monitoring specific marker molecules of corruption and deterioration	Ammonia and formaldehyde in fish	—	[11]
	Fructose and pectin in apple juice	Wavelets	[12]
	Protein oxidation/denaturation in beef	—	[13]

—: Not report.

**Table 2:** Label-free and label-based strategies for chemical contaminants detection by SERS in food.

Strategies	Recognition Elements	SERS Substrates	Distribution	Ref.
Label-free method	C≡N bond	Au NPs	Orange peels	[18]
	C–N	Poly(ethylene terephthalate)/indium tin oxide/Ag platform	Apple peels	[25]
	N–O bond	Ag NPs	Water	[19]
	O–S–O bond	Ag NPs	Wines	[29]
	O–S–O bond	Sandwiching the ZnO-paper disc	Wines	[30]
Label-based method	Probe molecules	Ag NPs	Water	[20]
	Modified substrates	Au nanorod-incorporated melamine foam	Chili sauce, dried chili, and chili powder	[21]
	aptamers	PCR sealing membrane	Cabbage	[22]

**Table 3:** Label-free and label-based strategies for mold and its mycotoxins contamination detection by Raman/SERS in food.

Strategies	Recognition Elements	SERS Substrates	Ref.
Label-free method	Crystal violet (CV) assay	AuNPs	[31]
	D_2_O	—	[32]
Label-based method	Antibody/immunoassays	Antigen-modified silica photonic crystal microspheres	[33]
	Aptamers	Silica photonic crystal microsphere modified AuNPs	[34]
	Molecularly imprinted polymers	Surface-Imprinted Gold Nanoparticle	[35]
	Linear polymer affinity agents	Poly(N-acryloyl glycinamide) polymers modified AuNPs	[36]

—: Not report.

**Table 4:** Label-free and label-based strategies for bacterial contaminations detection by Raman/SERS in food.

Strategies	Recognition Elements	Algorithms	SERS Substrates	Bacteria Species	Ref.
Spore	Dipicolinic acid	—	Gold nanoparticle-based substrates	*Bacillus anthracis*	[38]
Label-based method	Complementary DNAs	—	Au nanopillars	*Enterococcus faecium* and *Staphylococcus aureus*	[41]
	Aptamers	—	Dendritic porous silica nanoparticles-Au-MBA-aptamer	*Staphylococcus aureus*	[42]
Label-free method	—	Principal component analysis (PCA)	Au NPs	Seven foodborne bacteria (*Salmonella typhimurium* ATCC 50013, *Salmonella* O7HZ10, *Shigella boydii* CMCC51514, *Shigella sonnei* CMCC51529, *Shigella dysenteriae* CMCC51252, *Citrobacter freundii* ATCC43864, *Enterobacter sakazakii* 154)	[37]
	—	—	AgNPs	*E. coli* DSM 1116	[39]
	—	PCA	Ag/Si substrate	C. *Jejuni* NCTC 11351, C. *coli* ATCC 33559, C. *upsaliensis* ATCC 43954, C. *lari* ATCC BAA-1060, P. *aeruginosa* ATCC 27853	[40]

—: Not report.

**Table 5:** Label-free and label-based strategies for harmful metal contaminants detection by SERS in food.

Strategies	Recognition Elements	Harmful Metal Analytes	SERS Substrates	Ref.
Label-free method	—	Total As	Cu_2_O/Ag	[43]
	—	Trace Cd^2+^ ions	Flower-like Ag@CuO	[44]
Label-based method	Raman activity dye signal turn-on	Hg^2+^	Single-stranded modified-DNA AuNPs	[45]
	Raman activity dye signal turn-off	Hg^2+^	Methimazole-functionalized and cyclodextrin-coated silver nanoparticles	[46]
	Combined with other analytical technologies	Cd^2+^	Au@Ag core-shell nanoparticles	[47]

—: Not report.

**Table 6:** Label-based SERS detection in food allergens.

Allergens	Food Categories	Recognition Elements	SERS Substrates	Ref.
β-conglycinin	Soybean	Monoclonal antibody	AuNPs	[48]
Agglutinin	Soybean	Polymer	Metal film over nanosphere substrates	[49]
β-lactoglobulin	Milk	Aptamer	Au-Ag NanoUrchins	[50]

**Table 7:** Strategies of food freshness judgment by SERS methods.

Strategies	Analysis	Algorithms	Ref.
Combined with chemometrics	Honey	Convolutional neural network	[58]
	Olive oils	Integral ratio	[60]
Combined with specific target DNA	Spiking little duck meat in lamb roll, pork, beef, mutton, and steak samples	—	[59]
	Milk	—	[56]

—: Not report.

**Table 8:** Label-free and label-based strategies of SERS detection of genetically modified organisms.

Strategies	Analytes	Algorithms	Genetically Modified Organism Components	SERS Substrates	Ref.
Label-free SERS detection	Transgenic corn expressing specific *Bacillus thuringiensis* and *Agrobacterium* spp genes	Linear Discriminant Analysis	Chemical composition	—	[69]
Label-based SERS detection	*Bacillus thuringiensis* (Bt) gene-transformed rice	—	Bt gene	Au NPs	[67]
	Genetically modified organism soybean	—	Promoter, codon, and terminator	Au@Ag	[68]

—: Not report.

## Data Availability

No new data were created or analyzed in this study. Data sharing is not applicable to this article.
